# Bright and dark Talbot pulse trains on a chip

**DOI:** 10.1038/s42005-023-01375-x

**Published:** 2023-09-13

**Authors:** Jiaye Wu, Marco Clementi, Edgars Nitiss, Jianqi Hu, Christian Lafforgue, Camille-Sophie Brès

**Affiliations:** École Polytechnique Fédérale de Lausanne (EPFL), Photonic Systems Laboratory (PHOSL), STI-IEM, Station 11, Lausanne, CH-1015 Switzerland

**Keywords:** Integrated optics, Micro-optics, Silicon photonics

## Abstract

Temporal Talbot effect, the intriguing phenomenon of the self-imaging of optical pulse trains, is extensively investigated using macroscopic components. However, the ability to manipulate pulse trains, either bright or dark, through the Talbot effect on integrated photonic chips to replace bulky instruments has rarely been reported. Here, we design and experimentally demonstrate a proof-of-principle integrated silicon nitride device capable of imprinting the Talbot phase relation onto in-phase optical combs and generating the two-fold self-images at the output. We show that the GHz-repetition-rate bright and dark pulse trains can be doubled without affecting their spectra as a key feature of the temporal Talbot effect. The designed chip can be electrically tuned to switch between pass-through and repetition-rate-multiplication outputs and is compatible with other related frequencies. The results of this work lay the foundations for the large-scale system-on-chip photonic integration of Talbot-based pulse multipliers, enabling the on-chip flexible up-scaling of pulse trains’ repetition rate without altering their amplitude spectra.

## Abstract (web summary)

The generation of temporal Talbot effect, i.e., the formation of temporal self-imaging patterns, in integrated photonic devices, is limited by bulky setups that limit the repetition rate. The authors design a compact and tunable Talbot chip where the GHz-repetition-rate bright and dark pulse trains can be doubled without affecting their spectra.

## Introduction

Talbot effect, initially described by Henry Fox Talbot in 1836 as the spatial-interference-induced formation of self-imaging patterns^1^, has given rise to enormous interest for nearly two centuries due to its underlying physical mechanisms and potential applications. Since the invention of lasers and the implementation of modern ultrafast experimental techniques and instruments, there has been a rapid growth of the studies of the Talbot effect in the temporal^2–7^, spectral^8,9^, and azimuthal^10,11^ domains in the last decades^12^, which have consolidated the physical and mathematical understanding of this class of phenomena^13^. The temporal and spectral Talbot effects can be linked together through a space-time duality^14^, and it was recently found and theorised that all these domains of observations can be unified by the duality by isomorphism^15^, i.e., through space-time, position-momentum, and time-frequency dualities.

The temporal Talbot effect, in particular, can be fruitfully employed in many ultrafast applications, such as integer, fractional, and arbitrary repetition rate multiplication (RRM)^4,5,16^, broadband full-field invisibility^17^, and noiseless intensity amplification^18^. Among these, RRM is highly appealing, especially in the case of extra-cavity scenarios like optical communications^19^, passive amplification^20^, and microwave photonics^21^, where it can be implemented by spectral amplitude and/or phase filtering^2,3,5,7,12,22–24^ and provides a train of high-repetition-rate pulses that is hard to achieve in intra-cavity geometries. Besides the commonly studied temporal Talbot effect of bright pulse trains, the mixing Talbot patterns of dark pulse trains at higher RRMs was also recently investigated^7^. Also not long ago, a novel method, based on the combination of Mach-Zehnder interferometers (MZIs) and delay-line interferometers (DLIs), to generate temporal Talbot effects has been experimentally demonstrated^5^, shedding light on its suitability for scalable on-chip integration.

Realising photonic integration of optical functions, e.g., light sources (especially the well-established integrated microcomb sources)^25^, amplifiers^26^, or signal-processing components^27^, is a necessary step towards future applications and has attracted a huge amount of interest. Currently, the Talbot effects are observed and utilised on-chip in microscopy^28^, spectroscopy^29^, and Talbot-cavity integrated lasers^30–32^, serving as a coherent in-phase coupling element^31^. However, to the best of our knowledge, the on-chip generation of the temporal Talbot effect has rarely been discussed. One of the latest state-of-the-art demonstrations, for example, is spectral phase filtering using a waveguide Bragg grating on silicon^33^. Yet, the limited tunability of the Bragg grating does not allow fine-tuning to produce a higher output quality and leverage the full potential of Talbot photonic chips. Also, the demonstration of an integrated Talbot processor at a lower repetition rate than the reported 10 GHz is of significant importance. The amount of dispersion required—and hence the size of the device—scales indeed quadratically with the inverse of the repetition rate^7^, practically limiting the applicability of this approach to lower repetition rate scenarios. Moreover, the Talbot effect of the dark pulse trains has never been investigated in integrated optics to date. Manipulating bright and dark pulse trains with photonic integrated circuits (PICs), fully replacing complex and bulky combinations of instruments and equipment, is itself intriguing, enabling the possibility to seamlessly work with other PICs, and might pioneer potential all-optical system-on-chip (SoC) applications.

In this work, we design and experimentally demonstrate, for the first time to the best of our knowledge, a silicon nitride (Si_3_N_4_) Talbot PIC based on a cascaded MZI and DLI geometry. Unlike more conventional rate multiplier methods, where multiplying the repetition rate always leads to an alteration of the optical spectra, a key feature of the temporal Talbot effect is that the comb spectra could stay unaffected by energy redistribution while achieving integer multiplications of the repetition rate. The proof-of-principle PIC can imprint the temporal Talbot phase relation onto the spectral components of an in-phase optical comb and therefore produce the two-fold (2×) Talbot self-image, doubling the repetition rates of bright or dark pulse trains. This technology can be eventually scaled up to *N* delay lines, resulting in an *N*-fold self-image. A key advantage over the conventional dispersion-based method in terms of scalability is that, by the proposed MZI-DLI scheme, the required length of the delay lines scales linearly with the inverse of the repetition rate rather than quadratically, making this approach particularly accessible for lower repetition rate combs. In addition, embedded, electrically controlled thermo-optic actuators enable the chip to arbitrarily switch between a pass-through or a 2× RRM output. In principle, it could also be designed for other repetition rates by changing the length of the delay line. We believe that the results of this work could serve as a reference design for large-scale photonic integration and broaden the understanding and the use of MZI on-chip devices.

## Results

### Principle of temporal Talbot effect in delay line structures

Controlling the repetition rate of a pulse train inside a laser cavity, especially when integer multiplications are desired, can be very difficult. Conventional approaches to realise a multiplied repetition rate reduce the frequency components at the cost of significantly changing the amplitude spectra, e.g., spectral amplitude filtering^22,34,35^. The Talbot effect offers a solution to preserve the amplitude spectral profile (and, in particular, the number of comb lines) by linearly acting only on the phase spectrum of the input comb. The temporal Talbot phase relation takes the form *ϕ*_*k*_ = *π*(*p*/*q*)*k*^2^, with *k* being the comb mode index (*k* = ± 1, 2, 3, . . . ) relative to the centre line (*k* = 0). *p* and *q* are mutually prime positive integers. By means of quadratic Gauss sums^36^, the phase of each self-image also satisfies the Talbot phase relation^15^:

1 $${\varphi }_{n}=-\pi \left(\frac{s}{q}{n}^{2}+c\right).$$

Here, the parameters *s* and *c* are related to *p* and *q* via Eq. (2), and *n* is the index of the pulse self-image (*n* = 0, 1, . . . , *N* − 1). We follow the notations from refs. ^15,36^ and denote [1/*a*]_*b*_ as the modular multiplicative inverse operation and $$\big(\begin{array}{c}a\\ b\end{array}\big)$$ as the Jacobi symbol. The relation between the parameters *s*, *c*, *p*, *q* can now be expressed as^7^:

2 $$\left\{\begin{array}{lll} s = \displaystyle 2\left[{\frac{1}{2p}} \right]_{q},& c = \displaystyle \frac{{q - 1}}{4} + \frac{1 - \left(\begin{array}{c}{p}\\ {q}\end{array}\right)}{2}, & {{{\mbox{if}}}}\,q \in {\mathbb{O}} \\ s = \displaystyle \left[{\frac{1}{p}} \right]_{2q},& c = \displaystyle - \frac{p}{4} - \frac{1 - \left(\begin{array}{c}{q}\\ {p}\end{array}\right)}{2},& {{{\mbox{if}}}}\,q \in {\mathbb{E}} \end{array}\right.$$

where ${\mathbb{O}}$ and ${\mathbb{E}}$ are, respectively, the odd and even integer sets. The phase results of Eq. (2) rely on the parity of *q*. One can easily show that when this aforementioned phase relation is satisfied, the spectral shape is not affected.

**Fig. 1:**
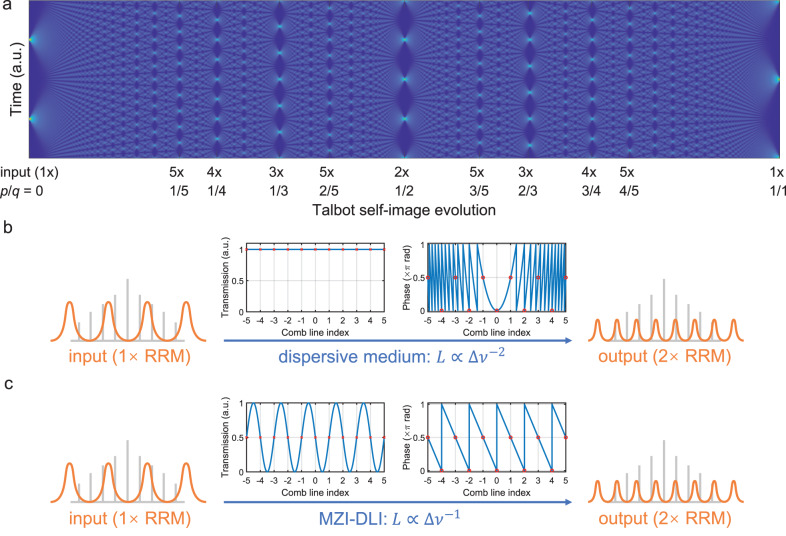
The mechanisms of temporal Talbot effect. **a** The temporal Talbot carpet. Here, the Talbot carpet shown is half of its full period, with marks for 1×–5× self-images. A non-phase-shifted 1× self-image will appear at *p*/*q* = 2/1. *p* and *q* are phase parameters described in Eq. (1). The 2× self-image at *p*/*q* = 1/2 is expected for both bright and dark pulse trains on the designed Talbot photonic chip, as theoretically and experimentally shown in Fig. 3c, f. The physical mechanisms of **b** the conventional dispersion-based and **c** the proposed Mach-Zehnder-interferometer (MZI)-based Talbot repetition-rate-multiplication (RRM) realisation. In the transmission and phase spectra, the solid blue lines are the theoretical curves, and the red circles are the points where the comb lines are.

By implementing the temporal Talbot phase relation, the evolution diagram of temporal profiles of an optical pulse train can be acquired, which is known as the Talbot carpet shown in Fig. 1a. For clarity and without loss of generality, Fig. 1a is a half-period demonstration. The other half period (*p*/*q* ∈ [1, 2]) is the mirror of Fig. 1a, with the cross-section (temporal profile) at *p*/*q* = 2 being exactly the same as at *p*/*q* = 0. The horizontal axis, *p*/*q*, can be understood as phase evolution or propagation distance, for it is also possible to launch a pre-assigned Talbot-phased optical comb into an SMF and allow phase accumulation through propagation^7^, which is illustrated in Fig. 1b. If a doubled repetition rate is desired, i.e., the 2× Talbot self-image, we seek the phase relation with *p*/*q* = 1/2 whose line-by-line phase relation of the comb should be [. . . *π*/2, 0, *π*/2, 0, *π*/2, 0, *π*/2, . . . ] according to Eq. (2).

It is reported that by incorporating the *N* parallel optical tapped delay line structure, the Talbot phase relation can be losslessly imprinted by re-distributing energy into each delay line with different designed lengths, resulting in *N*× RRM^5^. The key idea of this mechanism is to combine^24^ the effects of *spectral amplitude filtering*, which achieves *N*× RRM and *phase filtering*, which maintains the corresponding amplitude spectrum by altering the phase, to satisfy the temporal Talbot effect conditions. A schematic of this mechanism is shown in Fig. 1c. It is worth emphasising that the mechanism of this process is *not* as seemingly simple as just delaying the pulse long enough such that the delayed pulse train fits into the intervals of the non-delayed one when recombining and produces *N*× the repetition rate, as this operation would not, in general, satisfy the Talbot phase relation. On the contrary, the design for the phase is the key to preserving the amplitude spectra. After the energy splitting, the frequency components of the comb on each delay line accumulate a different phase during propagation. Due to the purely linear nature of the process, each frequency component remains unchanged and maintains perfect coherence with those in other delay lines. When they recombine, the line-by-line interference gives a phase relation that satisfies Eq. (1). This can be regarded as a combination of spectral amplitude filtering and spectral phase-only filtering^5^, and for the *N* = 2 Talbot PIC designed in this work by using the generalised Landsberg-Schaar identity^37^, the transfer function *H* of the combined filtering can be derived as:

3 $$H\left({f}_{k}=\frac{k}{2{\tau }_{{{{{{{{\rm{delay}}}}}}}}}}\right)\, 	= {\left.\frac{1}{N}\mathop{\sum }\limits_{n = 0}^{N-1}\exp \left(-2\pi n{f}_{k}{\tau }_{{{{{{{{\rm{delay}}}}}}}}}-i{\varphi }_{n}\right)\right| }_{N = 2}\\ 	 = \frac{1}{\sqrt{2}}\exp \left(-i\frac{\pi }{4}+i{\phi }_{k}\right)\\ 	 = \frac{1}{\sqrt{2}}\exp \left(-i\frac{\pi }{4}+i\frac{\pi {k}^{2}}{2}\right),$$

which is exactly [. . . *π*/2, 0, *π*/2, 0, *π*/2, 0, *π*/2, . . . ] for *k* = [. . . − 3, − 2, − 1, 0, + 1, + 2, + 3, . . . ]. Therefore, the *N* = 2 Talbot PIC is capable of imprinting the Talbot phase relation onto an input comb, doubling the repetition rate of the corresponding pulse train while keeping the amplitude spectrum unchanged.

It is worth noting that, as one can observe from Eq. (3), the imparted Talbot phase scales linearly with the time delay *τ*_delay_. By recalling the expression of the free-spectral range (FSR) for a generic DLI: Δ*ν* = *c*/(*n*_g_*L*), being *L* the arms unbalance and *n*_g_ the group index, we observe that, here, the length required scales linearly with the inverse of repetition rate (*L* ∝ Δ*ν*^−1^), rather than quadratically (*L* ∝ Δ*ν*^−2^) as in the case of dispersion-based temporal Talbot effect (see the form factor comparison in “Discussion”), making our approach intrinsically advantageous in the perspective application to low repetition rate combs.

### Chip design and characterisation

**Fig. 2:**
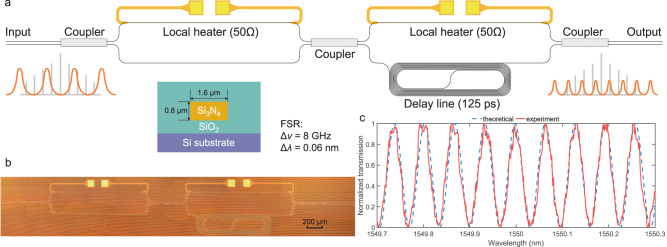
The Si_3_N_4_ Talbot chip. **a** Design schematic showing the components and the dimensions (cross-sectional view) of the photonic circuit; **b** Microscopic photograph of the chip; **c** Theoretical (blue dashed curves) and experimental (red solid curves) transmission spectrum of the device in the frequency multiplication configuration.

To realise the *N* = 2 tapped delay line structure on a chip, we designed a two-stage cascaded MZI configuration, in which the optical length of the reference arms can be tuned by electrically-driven thermo-optic actuators. A schematic diagram of the device is shown in Fig. 2a. The first-stage MZI ensures equal energy distribution into the second stage through local temperature control, while this control is also the key to the pass-through operation mode that allows a direct pass through the chip without manipulation. The second-stage MZI is a DLI with a delay line of 125 ps, corresponding to the half-period interval of a 4 GHz pulse train. The two branches are then recombined at a coupler, whereas the recombination phase is controlled by means of a thermo-optic actuator, which allows slight variations of the effective local index of the waveguide as a function of the bias current, ensuring the possibility of achieving the Talbot condition. A microscopic photograph of the device is shown in Fig. 2b.

A sinusoidal interferometric pattern exists owing to the existence of the unbalanced DLI. The transmission spectrum of the device in this configuration is shown in Fig. 2c. The FSR of the interferometer is designed to match the output comb repetition rate of 8 GHz (blue dashed line), as confirmed by the experimental measurement (red solid line). Note that the visibility of the interferogram fringes can be varied between 0 and 1 by acting on the first phase shifter. Similarly, the interferogram transmission function can be shifted horizontally by acting on the DLI local heater, thus allowing the tuning of the alignment of the input comb lines with the transmission function of the device.

### Proof-of-principle experiments: repetition rate doubling of bright and dark pulse trains

**Fig. 3:**
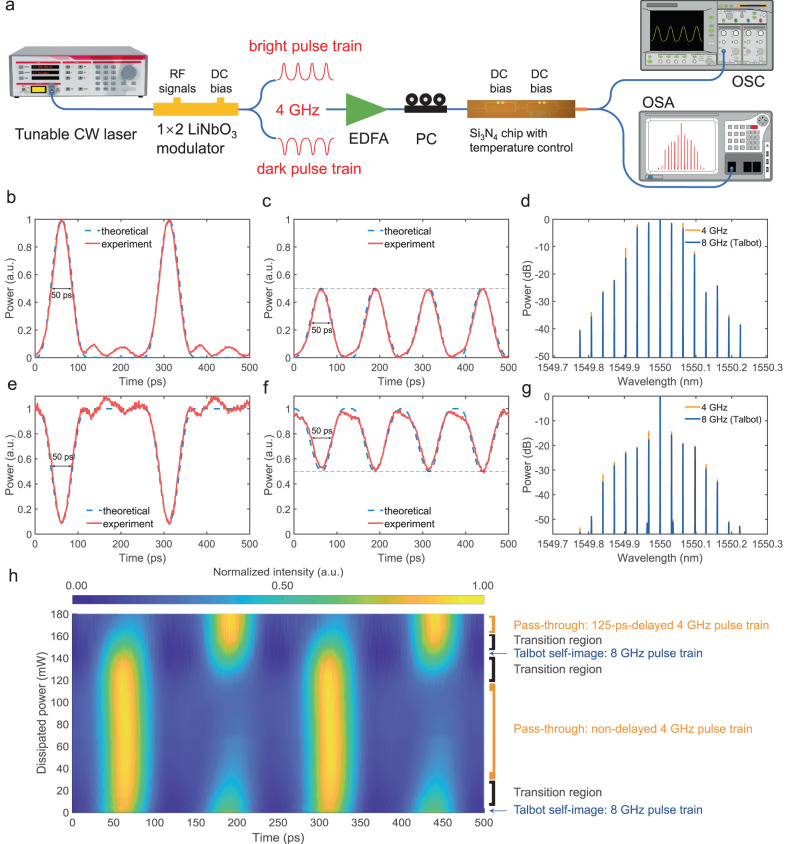
Temporal Talbot effect of bright and dark pulse trains on a chip. **a** Schematic of the experimental setup. CW continuous wave, EDFA erbium-doped fibre amplifier, PC polarisation controller, OSC oscilloscope, OSA optical spectrum analyser. Temporal profiles of **b** the original 4-GHz bright pulse trains at pass-through mode operation and **c** the corresponding 2× Talbot self-image of 8-GHz. Solid curves denote the experimental data, and the dashed curves represent the corresponding theoretical predictions. **d** Spectra comparison of these two pulse trains. **e**–**g** The corresponding temporal and spectral profiles of the dark pulse train. In (**b, c, e, f**), the theoretical curves are plotted in dashed blue lines, and the experimental ones are illustrated in solid red lines. In (**d** and **g**), the 4-GHz spectra are in yellow, and the 8-GHz (Talbot) spectra are in blue. **h** Temporal profile versus the dissipated power on the first-stage interferometer, showing the two operations of the chip and the transition regions in between.

We demonstrate the proof-of-principle experiments using the setup shown in Fig. 3a. We use a tunable 10 dBm continuous-wave (CW) laser at C-band as a light source. In light of the methods introduced in refs. ^10,38^, we utilise a harmonic combination of 4, 8, and 12 GHz sinusoidal waves to generate the 4 GHz modulation radio-frequency (RF) signal for the 1 × 2 lithium niobate (LiNbO_3_) Mach-Zehnder modulator (MZM). The MZM generates bright and dark pulse trains, respectively, at its two outputs, whose patterns are complementary to each other. Both of such input pulse trains have a full width at half maximum intensity (FWHM) of 50 ps. In the experiment, we connect to one output at a time.

The chip is mounted on a temperature-stabilised holder and set to work under a constant temperature of 25 °C. The local temperature of the MZI is also controlled by introducing a DC current through the embedded resistance. This local temperature change slightly affects the effective index and therefore the optical lengths of the reference arm of the first MZI, determining the light energy distribution into the second-stage DLI. By this control, the two modes of operation of this chip can be realised, namely, the pass-through mode, where the light goes through only one arm of the DLI (with negligible propagation loss), and the Talbot mode, where the light split evenly into the two arms of the DLI and recombined into a 2× Talbot self-image, which doubles the repetition rate.

The output signal is collected and analysed by a high-resolution optical spectrum analyser (OSA) and an oscilloscope (OSC) for the retrieval of its spectral and temporal profiles, respectively.

Besides the conventional bright pulse experiments, the designed Talbot chip could also work with dark pulse trains by the same principles. The results of the chip operations are illustrated in Fig. 3b–g. To the best of our knowledge, this is the first demonstration of the temporal Talbot effect of dark pulse trains on a chip, following the discovery and discussions on the mixing Talbot patterns of the dark pulse trains^7^. In Fig. 3b, e, by tuning the local temperature control on the first MZI, the pass-through mode of chip operation presents the exact same temporal profile as the input. Under this circumstance, the optical comb is still an in-phase comb without any manipulation. The measured pulse train fits the theoretical curves well.

By finely adjusting the DC voltage of the local temperature control, the DLI starts to provide a relative 125-ps (1/2 × 1/(4 GHz) = 125 ps) delay to one branch of the energy, and the chip can reach a state such that a relatively weaker secondary pulse train appears at the intervals of the original pulse train, finally stabilising at almost the same intensity. The created new pulse train has twice the repetition rate (8 GHz), as shown in Fig. 3c, f, which matches well with the theoretical 8-GHz pulse train in dashed lines. The measured FWHM of the bright 8-GHz pulse train is also 50 ps, while the FWHM of its dark counterpart is slightly smaller with a more un-even DC component between each two dark pulses, as can be interpreted from Fig. 3f. This is due to the destructive intra-pulse interference happening between the overlapping part of the two recombined pulse trains.

The spectral results are shown in Fig. 3d, g. The intervals between each comb line for the pass-through and the Talbot operations are both exactly 0.032 nm, corresponding to the 4-GHz frequency difference at the C-band. In this figure, the spectra of the pass-through operation mode are plotted in light orange, and those of the Talbot operation mode are plotted in dark blue. The output spectra fit each other quite well without any frequency component loss or bandwidth change, denoting the presence of the temporal Talbot effect, as discussed in the previous section. The small observable spectral discrepancy between the two operation modes is due to laser frequency and power jittering, as well as possible amplifier noises, which is not a part of the Talbot effect and can be neglected, making the comparison between the theoretical analyses and experimental results accurate. The preservation of the spectral profile could be further improved by SoC-level packaging, taking advantage of a well-established technology trend in integrated photonics.

We further experimentally investigate the temporal evolution of the 4 GHz pulse train into the 8 GHz one, as depicted in Fig. 3h for the bright pulse train and the chip temperature set to 25 °C. We record the dissipated electrical power on the first-stage local thermo-optic actuator while the DC voltage of the second-stage actuator is kept constant such that in the Talbot operation region, the chip produces an 8 GHz pulse train with equal intensity and unchanged spectrum. The dissipated power is monotonously linked to the effective phase shift, therefore, by measuring the dissipated power, we are equivalently following the evolution of the effective phase shift. It can be observed that the designed Talbot PIC works in Talbot operation mode within the small vicinity of 0 mW. When the DC power increases, the intensity of the secondary pulse train gradually vanishes. This is marked by the transition region in Fig. 3h. From 40 to 120 mW, the chip operates at the pass-through mode, producing the original 4 GHz pulse train. Near 150 mW, the 2× Talbot self-image of the optical pulse train emerges again. For higher DC power, the chip is in pass-through mode but with a 125-ps delay with respect to the aforementioned pass-through mode region. This also proves that in the pass-through mode, the energy indeed flows mainly through one arm of the second-stage MZI. The power of 180 mW is the upper limit we set for the chip in order to protect it from potential damage. Therefore, Fig. 3h shows a nearly complete operation period of the Talbot PIC, giving a clear picture of its operating mechanism.

**Fig. 4:**
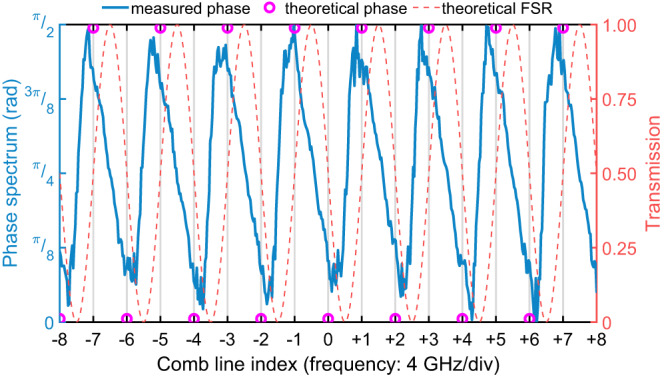
The phase of each comb line of the output at Talbot operation. The measured phase is extracted from the free-spectral range (FSR) data shown in Fig. 2c. The grey vertical grid shows the spectral locations of each comb line with a 4 GHz separation. The red dashed line is the theoretical FSR, the purple circles are the theoretical phase, and the blue solid line denotes the experimental phase data.

To further investigate the link between our observations and the Talbot phase relation expressed by Eq. (3), we estimated the phase imparted to each comb line by reconstructing the phase transmission spectrum of our device when operated in the Talbot configuration. Such calculated phase spectrum, shown in Fig. 4, was inferred by exploiting the analytic relation between the real and imaginary parts of the device transfer function. In particular, the trace was obtained numerically from the amplitude transmission spectrum shown in Fig. 2c by a Hilbert transform^39^. Similar to the widely used Kramers-Kronig relations, this method is based on the principle of causality, leading to an analytical relation between the real and imaginary parts of the transfer function. This allows the extraction of the phase from the transmission spectrum. The grey vertical lines represent the spectral location of the comb lines with respect to the transmission spectrum, and the purple circles are the theoretical Talbot phase calculated by Eq. (1). As expected, the comb lines experience a nearly [. . . *π*/2, 0, *π*/2, 0, *π*/2, 0, *π*/2, . . . ] phase shift, confirming the matching of the Talbot phase relation in our experiment. The small deviations between the measured phase and the theoretical one are due to the limited wavelength control resolution of the laser used for measuring the transmission spectrum (Fig. 2c).

In principle, the designed Talbot PIC can work as a broadband processing device, the main limiting factor being the 2 × 2 embedded multi-mode interference (MMI) couplers, whose nominal 3 dB bandwidth is around 40 nm. For input wavelengths outside this band, the MMI couplers splitting ratio is altered, resulting in an imbalance in the MZI splitting and recombination ratios. Besides, the DLI acts like a double-edged sword. On the one hand, it provides the essential delay to realise Talbot effect, but on the other hand, when operating on ultrashort pulses with a broad spectrum, additional dispersion compensation might be needed to counteract distortion.

## Discussion

**Fig. 5:**
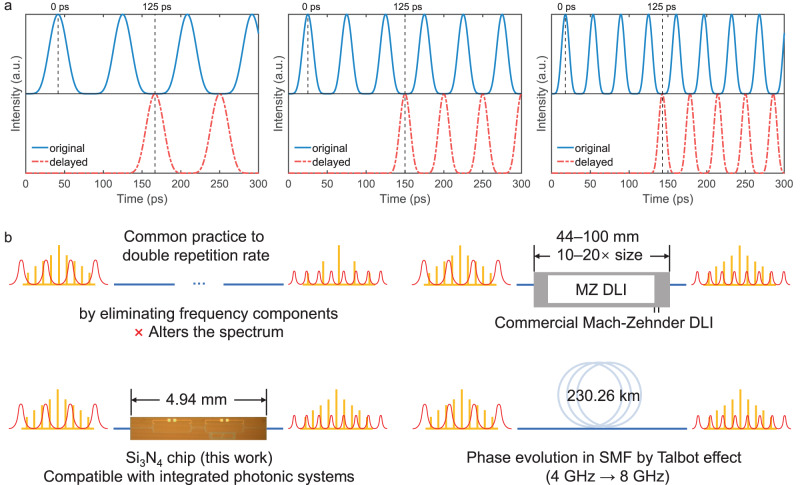
Comparisons with other operation frequencies and different solutions. **a** Illustration of the compatibility with other operating frequencies, with the examples being 12, 20, and 28 GHz inputs and forming the corresponding 24, 40, and 56 GHz outputs. The delay between the two vertical dashed lines is 125 ps, as in the chip design. The blue solid lines denote the original pulse trains, and the red dash-dotted lines represent the delayed pulse trains. **b** Form factor comparison among the other solutions to double the repetition rate of a pulse train. The schematic sketch of the waveforms (red) is in the temporal domain, and the comb spectra (yellow) are in the frequency domain. MZ DLI Mach-Zehnder delay line interferometer, SMF single-mode fibre.

The proposed chip design can not only work with the standard 4-GHz pulse train but also with any other repetition frequencies that are the odd multiples of 4 GHz, i.e., 12 GHz, 20 GHz, 28 GHz, etc. Limited by the technical specifications of the RF components, here we discuss theoretically the possibility to operate at these frequencies. The illustrations in Fig. 5a show intuitively how and when the two trains of pulses align to form the doubled repetition rate. The repetition rates of the pulse trains in Fig. 5a are 12 GHz, 20 GHz, and 28 GHz, respectively, and the first pulses in each panel can be regarded as the very first input pulse.

The repetition rate requirement, *f*_*r*_ = 1/*t*_rep_, for the designed chip to work is *ξ*/2 ⋅ *t*_rep_ = *τ*_delay_ = 125 ps, with *ξ* being an odd number, as for even values, the delayed pulse train will overlap with the non-delayed one. This simple rule ensures that the delayed pulse train falls precisely in the middle of the original pulse intervals stably. Furthermore, the temporal Talbot phase relation, as shown in Eq. (3), is still satisfied as it is independent of the input repetition rate. Theoretically, at a given carrier wavelength (e.g., in the C-band), the only physical limitations for higher frequencies are pulse generation and temperature control accuracy since the DC power region of Talbot operation might vary. However, the latter can be better controlled in fully integrated platforms.

From an engineering perspective, the length of the delayed arm of the second MZI could be extremely long if the design is altered for a much lower frequency (e.g., in the regime of MHz), which can cause a large amount of propagation loss. Fortunately, the state-of-the-art SiN photonic platform provides a <0.2 dB ⋅ cm^−1^ propagation loss^25^, making it possible to realise even metre-long delay lines which can work with significantly lower frequencies than the demonstrated 4 GHz.

As for the scaling to higher RRMs, the challenge lies in concatenating multiple levels of MZI-DLI interferometers, either in series (cascading) or in parallel^5^. Each level needs to be controlled separately, and due to the potential error and non-uniformity in fabrication, the DC power control can be different from level to level. For higher RRM, the size of the chip grows accordingly, and the system complexity also increases with the RRM factor *N*. A careful device engineering, together with new cross-disciplinary technologies—such as computer-controlled self-tuning enabled by machine learning^40^—may be useful in such high RRM applications.

It is intuitive to think that performing Talbot operations in the designed device is not a lossless process due to interference. If we consider the chip to be a one-input one-output device, for an input *K*-line optical comb, the total power of the source should be ${P}_{{{{{{{{\rm{in}}}}}}}}}\propto {\left\vert {E}_{0}\right\vert }^{2}{K}^{-1}{\sum }_{k}\left\vert {A}_{k}\right\vert$ with *k* = 0, 1, . . . , *K* − 1 and *A*_*k*_ being the envelope of the signal. Due to the MZI interference, the total power at the output should be ${P}_{{{{{{{{\rm{out}}}}}}}}}\propto {\left\vert {E}_{0}\right\vert }^{2}{(NK)}^{-1}{\sum }_{k}\left\vert {A}_{k}\right\vert$ which is half of the original input comb (*N* = 2). This is exactly what happened in the proof-of-principle setup shown in Fig. 3a since the focus lens could only pick up one of the two outputs at a time. In this single-output implementation, the loss scales with 1/*N*, which is non-negligible for higher *N* (e.g., 10 dB for *N* = 10 and 30 dB for *N* = 1000).

However, the efficiency can reach nearly 100% (without taking into account the chip coupling loss) if both of the two outputs are collected. The output coupler can be regarded as a 2 × 2 discrete Fourier transform star network^41^, which provides two 2× RRM Talbot pulse trains^5^, which may be beneficial for applications such as optical clock distribution among many components. It should be noted that the two output channels are coherent with each other, carrying exactly the same optical signal up to a constant phase factor. Consequently, they can be recombined at a 50:50 directional coupler, recovering the ideally lossless operation that is a peculiarity of Talbot effect. In other words, the total output power of the system is preserved among all the output modes; these can be ideally recombined whenever a single-output mode and low loss are required.

When our design is integrated with other optical functional components at an SoC level, the chip coupling loss can be greatly reduced, and the two outputs can be more efficiently utilised. This again emphasises the importance of miniaturising macroscopic optical setups and integrating them onto a PIC.

Another main characteristic of this design is its form factor, made possible by photonic integration in silicon nitride technology. To assess the potential and practicality in future applications, we compare the sizes of approaches for repetition rate doubling, which are plotted in Fig. 5b. In addition to the conventional split and delay practice, which affects the spectrum, there are several methods to assign the Talbot phase relation to each frequency line of an optical comb.

The tapped delay line structure can be realised by commercial MZIs and DLIs with conventional centimetre- to decimetre-scale components. The system is therefore 10–20 times larger than the 4.94 mm Talbot PIC and can be more expensive owing to a lower wafer yield. Additionally, they are not suitable for SoC-level integration or seamlessly working with other PICs. Another solution could be assigning the Talbot phase line-by-line using programmable wave-shapers (pulse-shapers)^7^. However, these devices are currently bulky (>20× larger in their longest dimension) and less likely to be scaled down and integrated. More importantly, they can be limited by the resolution of their pixels when working with low repetition rate combs.

The aforementioned SMF propagation method is simple and relatively convenient when the repetition rate of the pulse train is high. This technique allows the pulse train to naturally evolve to the *p*/*q* = 1/2 state by dispersion-induced accumulated phase, as shown in Fig. 1b. However, when it comes to low repetition rate, the length of the SMF needed can be impractically long^13^ according to *L* = 2*πp*/(*q*∣*β*_2_∣Δ*ω*^2^). For a brief comparison, here we take the second-order dispersion (group velocity dispersion) *β*_2_ = −21.6 ps^2^km^−1^ for silica SMF. The required length for the 4-GHz pulse train at *p*/*q* = 1/2 is 230.26 km, which is 4.6 × 10^7^ times longer than a chip and would likely display impractically high propagation losses. The lower the frequency is, the greater the advantage of going on-chip.

On the PIC level comparison, the current state-of-the-art Talbot chip using Bragg grating waveguide measures a length of ≈8 mm for 2× RRM at 10 GHz input, while for linearly chirped waveguide Bragg gratings, it would be ≈4 cm (c.f., ref. ^33^). These values are respectively 1.6× and 8.1× larger than our proposed design, that also operates at a much lower repetition rate of 4 GHz. If the previously reported state-of-the-art designs were to be scaled for the same 4 GHz repetition rate we demonstrate, their devices should be made >4× larger.

## Conclusions

In this work, we propose, design, and experimentally demonstrate a PIC with cascaded MZIs that converts in-phase optical combs into Talbot-phased ones with their repetition rates doubled while keeping their spectra unaltered. The chip is compatible with both bright and dark pulse trains and works with a variety of different repetition rates. The embedded temperature control also allows the chip to work in a pass-through mode, such that switching of the functions of this chip is readily realised. The evolution of the temporal profiles from the original input to the 2× Talbot self-image is analysed. Additionally, we show, for the first time, to the best of our knowledge, the temporal Talbot effect of dark pulse trains on a chip. On-chip makes our device more compact than other repetition rate doubling solutions. We believe that the results of this work could be very useful in the applications of on-chip Talbot effects and could provide a deeper insight into the physics of this phenomenon. The results discussed in this work also have the potential to be applied in optical communications, amplification, and imaging.

## Methods

### Chip design

The chip was fabricated through a multi-project wafer run at a commercial foundry (LIGENTEC SA). The Si_3_N_4_ waveguides have a dimension of 1.6 × 0.8 μm, encapsulated in the silicon dioxide (SiO_2_) layer on top of the silicon (Si) substrate, as shown in the cross-sectional view of Fig. 2. According with simulations, the fundamental transverse electric (TE) mode has an effective refractive index of 1.6769 at 1550 nm. The separation between the two parallel waveguides is 22 μm, while the coupler structures enable a 50:50 power splitting and therefore a continuously tunable splitting ratio at the output of the first-stage MZI. The second-stage MZI is a DLI with a delay line corresponding to the half-period interval of a 4 GHz pulse train (125 ps). The electrical resistance of the two thermo-optic phase shifters is ≈50 Ω.

### Pulse train generation and instruments

The RF signals are generated by an Anritsu MG3692C, an Agilent E8257D, and an Agilent MXG N5183A. The RF amplifier is a ZVA-0.5W303G+ working with a 10 MHz–20 GHz frequency range from Mini-Circuits. The MZM LiNbO_3_ intensity modulator is an AX-1x2-0MSS-20-PFA-PFA-LV from EOSPACE. The pulse train is generated by a quasi-Nyquist method adapted from ref. ^38^. The EDFA is an Optilab EDFA-16-LC-M. The tunable laser source is Yenista Tunics-T100S-HP. The chip is mounted on a custom stage with a Peltier array and sensors, monitored and controlled by a Thorlabs TED200C temperature controller, and set to stabilise at 25 °C. The spectra are collected by an APEX AP2043B OSA, and the temporal profiles are measured by an Agilent Infiniium DCA 86100A wide-bandwidth oscilloscope with an Agilent 86105A 20 GHz optical module.

## Data Availability

The data that support the plots within this paper are available at 10.5281/zenodo.8272168.

## Code availability

The codes used to produce the results of this paper are available at 10.5281/zenodo.8272168.
